# Discriminative local subspaces in gene expression data for effective gene function prediction

**DOI:** 10.1093/bioinformatics/bts455

**Published:** 2012-07-20

**Authors:** Tomas Puelma, Rodrigo A. Gutiérrez, Alvaro Soto

**Affiliations:** ^1^Department of Molecular Genetics and Microbiology, FONDAP Center for Genome Regulation, Millennium Nucleus Center for Plant Functional Genomics and ^2^Department of Computer Science, Millennium Nucleus Center for Plant Functional Genomics, Pontificia Universidad Catolica de Chile, Santiago, Chile

## Abstract

**Motivation:** Massive amounts of genome-wide gene expression data have become available, motivating the development of computational approaches that leverage this information to predict gene function. Among successful approaches, supervised machine learning methods, such as Support Vector Machines (SVMs), have shown superior prediction accuracy. However, these methods lack the simple biological intuition provided by co-expression networks (CNs), limiting their practical usefulness.

**Results:** In this work, we present Discriminative Local Subspaces (DLS), a novel method that combines supervised machine learning and co-expression techniques with the goal of systematically predict genes involved in specific biological processes of interest. Unlike traditional CNs, DLS uses the knowledge available in Gene Ontology (GO) to generate informative training sets that guide the discovery of expression signatures: expression patterns that are discriminative for genes involved in the biological process of interest. By linking genes co-expressed with these signatures, DLS is able to construct a discriminative CN that links both, known and previously uncharacterized genes, for the selected biological process. This article focuses on the algorithm behind DLS and shows its predictive power using an *Arabidopsis thaliana* dataset and a representative set of 101 GO terms from the Biological Process Ontology. Our results show that DLS has a superior average accuracy than both SVMs and CNs. Thus, DLS is able to provide the prediction accuracy of supervised learning methods while maintaining the intuitive understanding of CNs.

**Availability:** A MATLAB® implementation of DLS is available at http://virtualplant.bio.puc.cl/cgi-bin/Lab/tools.cgi

**Contact:** tfpuelma@uc.cl

**Supplementary Information:** Supplementary data are available at http://bioinformatics.mpimp-golm.mpg.de/.

## 1 INTRODUCTION

Discovering the biological processes that genes carry out inside the cell is a major challenge to understand gene function at a genome-wide scale. Unfortunately, many organisms lack in-depth understanding about the genes involved in specific biological processes. As an example, in the favorite model in plant biology, *Arabidopsis thaliana*, 16 319 (52%) of its genes lack annotations about their biological processes in the Gene Ontology (GO) database (GO annotations date: November 9, 2010) (Ashburner *et al.*, 2000; http://www.geneontology.org).

Machine learning (Mitchell, 1997) has emerged as one of the key technologies to support gene function discovery. In particular, many methods have been proposed to take advantage of the massive amounts of microarray expression data available (see Valafar, 2002; Zhao *et al.*, 2008 for reviews). These prediction methods can be classified into two broad groups: supervised and semi-supervised approaches. On one hand, supervised techniques use a labeled training set of genes to learn how to discriminate the genes of each label or function. On the other hand, semi-supervised approaches first group genes in an unsupervised manner, without using any functional information, and then a prediction is performed, usually by propagating the over-represented functions among the genes of each group (‘guilt-by-association’ rule, Walker *et al.*, 1999).

Among supervised machine learning techniques, Support Vector Machines (SVMs) (Cortes and Vapnik, 1995) have been one of the most successful approaches to predict gene function, as has been shown by several works (Brown *et al.*, 2000; Mateos *et al.*, 2002; Yang, 2004; Barutcuoglu *et al.*, 2006). However, despite their theoretical advantage in terms of classification accuracy, in practice, SVMs present the mayor inconvenience of operating as a black-box (Barakat and Bradley, 2010). Although additional techniques can be applied to extract comprehensible semantic information from SVM models, their application is not straightforward and is usually restricted to linear-SVM models (Guyon *et al.*, 2002; Fung *et al.*, 2005; Wang *et al.*, 2009). In the general case of non-linear SVMs, the transformation of the data to high-dimensional spaces complicates any interpretation of the SVM solution. In our experience, this is a major limitation for gene function discovery as understanding the predictions is a key aspect to evaluate their biological soundness and guide research. This aspect is even more critical considering the incomplete nature of annotations and the capability of genes to have multiple functions, which prevents obtaining an error-free gold standard, and thus evaluating the absolute accuracy of the methods (false-negative problem; Mateos *et al.*, 2002; Jansen and Gerstein, 2004).

In contrast to supervised methods, many semi-supervised approaches have emerged based on simpler, but biologically sound concepts, such as co-expression and the ‘guilt-by-association’ rule (Eisen *et al.*, 1998; Walker *et al.*, 1999; Kim *et al.*, 2001; Stuart *et al.*, 2003; Blom *et al.*, 2008; Horan *et al.*, 2008; Vandepoele *et al.*, 2009; Lee *et al.*, 2010; Ogata *et al.*, 2010; Bassel *et al.*, 2011). The basic assumption in these methods is that if a group of genes shows synchronized (correlated) expression patterns, then there is a high chance for them to participate in a common biological process. Common techniques used to group genes are clustering (Eisen *et al.*, 1998; Alon *et al.*, 1999; Horan *et al.*, 2008), biclustering (see Madeira and Oliveira, 2004; Tanay *et al.*, 2005; Prelić *et al.*, 2006 for reviews) and co-expression networks (CNs; Stuart *et al.*, 2003; Vandepoele *et al.*, 2009; Bassel *et al.*, 2011).

Unfortunately, current methods based on CNs do not offer the accuracy of supervised methods to predict gene function, as we show in this work by comparing the performances of CNs and SVMs. Furthermore, their classification strategy poses some relevant inconveniences. In particular, the selection of a suitable correlation threshold to define co-expressed genes is often difficult and arbitrary. Furthermore, both CNs and clustering rely on global co-expression patterns, meaning that genes need to be co-expressed in a large proportion of the data in order to be grouped together. Usually, these data involve hundreds or thousands of microarray experiments, each measured under a wide range of experimental conditions, such as different time points, tissues, environmental conditions, genetic backgrounds and mutations. In this scenario, expecting global co-expression becomes a strong imposition and limitation.

The previous observation has motivated the development of biclustering algorithms (Cheng and Church, 2000). The main idea behind biclustering is to find clusters of genes that co-express in subsets of experimental conditions. After the seminal work by Cheng and Church (2000), an extensive list of biclustering approaches has been developed (see Madeira and Oliveira, 2004; Tanay *et al.*, 2005; Prelić *et al.*, 2006 for reviews). However, besides their theoretical advantages, these approaches have not been extensively used in practice. Based on our experience, the unsupervised local search of experimental conditions often leads to clusters with genes from a broad range of functions, thus, limiting their discriminative properties. This problem is even worse considering the noisy nature of microarray data, which often leads to the discovery of biologically meaningless biclusters. Selecting datasets in a ‘condition-dependent’ fashion should more precisely identify gene interactions relevant to a specific biological question at hand (Bassel *et al.*, 2011). However, given the amount of expression data available today, manual selection of the relevant conditions is not a practical solution in most cases.

To overcome the state of the art limitations exposed above and aid gene functional research, we present Discriminative Local Subspaces (DLS), a novel machine learning method that discriminatively predicts new genes involved in a biological process of interest by building a discriminative CN. DLS takes advantage of the discriminative nature of supervised learning while maintaining the expressiveness of CN approaches.

Unlike other co-expression-based methods, DLS exploits the existing knowledge available in GO to construct informative training sets. These training sets guide the search of suitable subsets of experimental conditions containing *expression signatures*. An expression signature corresponds to a discriminative expression pattern with two key properties: (i) it is defined in a local subspace of the data (i.e. a particular gene and a subset of experimental conditions) and (ii) it is highly discriminative (exclusive) for the positive training genes associated to a biological process of interest. As a further feature and to tackle the inherent noise of negative training sets (genes not related to a biological process), DLS incorporates a procedure that iteratively predicts false-negative (FN) genes and refines the training set in order to improve its prediction performance.

The discriminative nature of expression signatures allows DLS to reveal novel co-expression associations for the selected process. In contrast to discriminative black-box models, such as SVMs, these predicted associations can be exposed in the context of a discriminative CN, giving the scientist the possibility to visualize, evaluate and interpret the predicted associations.

Unlike traditional CN, DLS does not rely on a predefined and fixed correlation threshold to construct the networks. Instead, DLS uses a Bayesian probabilistic approach that adaptively derives a confidence score for each predicted association. A network is then constructed based on a desired minimum confidence, which is translated into different correlation thresholds depending on the discriminative level of each signature.

In order to test the prediction power of our method, we use an *A. thaliana* expression dataset containing 2017 microarray hybridizations. We compare DLS performance with respect to CN and two versions of SVM, linear-SVM and radial basis kernel (RBF)-SVM. The accuracy and predictive power of the methods are tested using cross-validation and also testing the enrichment of year 2008 predictions with respect to new 2010 annotations, using 101 representative GO terms from the Biological Process Ontology. Our results reveal that DLS attains superior average accuracy and similar predictive power than RBF-SVM. Furthermore, they show a clear advantage for DLS over linear-SVM and CN in both tests. Remarkably, they show that unlike SVM and CN, DLS is able to systematically improve its predictive power when increasing the number of available experimental conditions.

The rest of the article presents the details behind DLS method (Section 2), our experimental setup (Section 3), the main results (Section 4) and our principal conclusions of this work (Section 5).

## 2 METHODS

DLS consists of four main consecutive steps: pre-processing of raw data, construction of a labeled training set, training and classification (or prediction). Additionally, DLS has two relevant steps for gene function prediction: the construction of a discriminative CN of predictions and the discovery of potential FNs in the training set. We detail next each of these main parts that compose the proposed DLS method.

### 2.1 Expression data pre-processing

A key aspect to use massive microarray data to perform effective gene functional predictions is to apply suitable pre-processing steps to extract informative features and to handle the noisy nature of raw expression data. We consider a generic case, in which we have a dataset containing multiple microarray experiments, each performed in replicates among several experimental conditions and coming from different sources. We organize this dataset in *M* control–test pairs of experimental conditions. These pairs can be manually defined by an expert or by using the automatic procedure described in the following paragraph. For each defined pair, we apply the RankProducts algorithm (Breitling *et al.*, 2004), which provides a statistical methodology to find the significance level between expression changes of genes over two experimental conditions with replicates. From this procedure, we obtain a 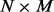 matrix *X*_LR_ with *N* genes and *M* log-ratio expression features, each corresponding to the logarithm of the fold change between the gene expressions in the test with respect to the control condition. The statistical significance of each change is provided in a second *N* × *M X*_FDR_ containing false discovery rates (FDRs). In few words, a small FDR value indicates that the corresponding change has a highly consistent rank among the replicates of the compared experiments and thus a low probability of being a false-positive detection (Breitling *et al.*, 2004). The *X*_FDR_ matrix is used by DLS to guide the search of discriminative expression pattern in *X*_LR_, by favoring the features with significant expression changes. A schematic view of this process can be seen in Supplementary Figure S1.

Manual definition of control–test pairs of experimental conditions can be a tedious and time-consuming task when using public databases containing thousands of microarray slides. Unfortunately, few public databases provide well-formatted annotations and labels for the available slides. Thus, in most cases it is impossible to systematically find the control–test pairs of conditions originally defined for each experiment. However, in many cases, it is possible to define which slides are replicates and which are part of the same experimental set. Thus, we propose an automatic procedure that uses this information in order to generate all possible pairs of conditions within a given experimental set, generating one log-ratio feature vector for each of them using the RankProducts method. Thus, if an experiment has *N*_c_ different conditions, our procedure generates *N*_c_ (*N*_c_ − 1)/2 log-ratio expression features. In order to minimize the redundancy that this procedure might generate, we consider a feature vector only if it does not have a ‘high’ correlation with any of the already added features of the same experiment. In the dataset used in this work, we define as ‘high’ a correlation >0.9. Although this procedure might generate some biologically meaningless comparisons, they should not affect the performance of DLS because its automatic selection of discriminative features should filter non-informative features. Moreover, even if some unexpected informative comparisons are found, these may provide new biological insights about the predictions and the process.

### 2.2 Training set: acquisition of functional labels

In order to search for discriminative expression patterns for a specific Biological Process of interest (BP), DLS needs a labeled training set of genes. Each training gene must be labeled as positive or negative, depending on whether the gene participates or does not participate in *BP*, respectively. DLS derives these labels using the gene annotations available in GO (Ashburner *et al.*, 2000). These annotations are organized hierarchically as a directed acyclic graph (DAG) of functional terms, going from the most general term, at the root node, down to the most specific terms, at the leaves of the graph. A relevant fact of this hierarchical organization is the upward propagation of functional annotations. More precisely, genes that receive a direct annotation at a specific level of the hierarchy also inherit all the functional annotations of their more general ancestors in the hierarchy.

The derivation of positive class 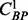 consists of selecting the genes annotated directly or by inheritance in GO terms related to *BP*. Optionally, this list can also be customized by the user. The derivation of the negative class 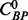 is a more ambiguous task, mainly due to missing functional labels. In effect, the list of annotations in GO is still incomplete, therefore it does not preclude that a gene not annotated with a particular biological process might indeed participate in it. Furthermore, the almost total absence of negative annotations and the ability of genes to be involved in multiple biological processes add extra complications. We face these inconveniences by using the multiple GO annotations of the positive genes to build a set 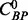 composed of genes that have a ‘low chance’ of being involved in *BP*. Our main intuition is that GO terms containing a substantial number of positive genes are likely to be functionally related to *BP*, and hence, they have a high chance to contain genes involved in *BP*. Following this intuition, we consider a GO term as ‘negative’ if it contains no more than a percentage *P* of genes already included in 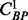. Consequently, the negative training set 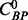 is formed by genes that have at least one direct annotation in a ‘negative’ GO term and do not have annotations in positive (non-negative) GO terms. According to our experiments, a value of *P* = 5% provides a good trade-off between the rates of false and true negatives. To handle the case of mislabeled genes, DLS also incorporates a false negatives discovery option that helps to refine the training set (details in Section 2.6).

### 2.3 Training: identifying expression signatures

The aim of the training scheme used by DLS is to identify a set of suitable expression signatures for the biological process of interest *BP*. Each expression signature is defined by a discriminative local subspace of the expression data matrix *X*_LR_ described in Section 2.1. The core of this scheme is based on four concepts about gene expression:

1. Co-expression: genes exhibiting co-expression patterns are likely to be co-regulated, and hence, they are likely to participate in a common biological process. Consequently, DLS uses the positive genes 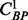 to search for characteristic co-expression patterns for genes involved in *BP*.
2. Subspaces: genes participating in the same biological process are usually not co-regulated under all cellular conditions. Consequently, DLS searches for co-expression patterns among subsets of experimental conditions.
3. Discrimination: genes not sharing a common biological process may co-express under some experimental conditions. Consequently, DLS uses the negative genes 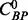 to filter out non-discriminative subsets of conditions where positive and negative genes show co-expression patterns.
4. Locality: genes participating in the same biological process might be regulated by different transcription factors and hence, they might co-express under different experimental conditions. Consequently, DLS independently searches for a suitable subset of discriminative conditions for each positive gene in 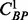.

In agreement with the previous concepts, the core of the training process consists of a feature selection algorithm that looks for a suitable expression signature for each gene 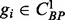. We achieve this by selecting a subset of features where 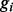 shows ‘strong’ co-expression with genes in 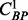 and ‘weak’ co-expression with genes in 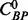. This feature selection algorithm explores the space of possible subsets of features using the Expression Signature Score (ESS) presented in equation (1). This score evaluates the discriminative power of each potential subset (pattern). Once the feature selection scheme is finished, each positive gene 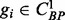 has an associated subset of features 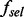 corresponding to the most discriminative expression pattern found by DLS. However, only expression patterns having an 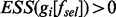 are selected as valid expression signatures and used in the classification process. We describe next the details of the ESS score and then the main steps behind the operation of the feature selection scheme.

#### 2.3.1 Expression Signature Score

Let vector 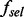 be a subset of the total set of available features. Furthermore, let 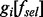 be the expression pattern of gene 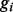 considering only the features in 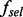 (i.e. 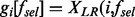). The ESS of gene 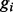 for a subset of features 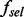 is defined as
(1)
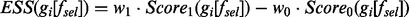

where 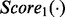 and 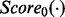 are functions that quantify the level of co-expression of gene 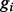 with respect to the set of genes in 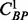 and 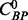, respectively, considering only features in 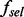. More precisely
(2)
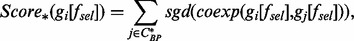

where 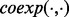 measures the co-expression between two patterns and 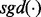 corresponds to a sigmoidal function used to establish a continuous threshold to separate ‘strong’ from ‘weak’ co-expressions. The shape of this sigmoidal function was tuned for best performance for function prediction using tests described in Section 3 and taking into account our biological and mathematical knowledge (Supplementary Fig. S2). As a result, the function returns values between 0 and 1, being close to 0 for co-expressions with values below 0.6 (weak) and above 0.5 for co-expressions above 0.8 (strong).

To measure co-expression between the expression patterns of two genes 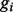 and 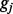 considering features in 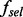, we use the absolute value of the cosine correlation, which can be expressed as the dot product of two vectors, normalized by their respective magnitudes:
(3)
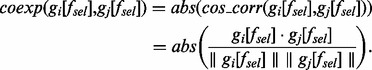



The cosine correlation (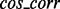) returns a continuous value between 1 and −1, taking a value of 1 if the two patterns are correlated, −1 if they are negatively correlated and 0 if they change independently. We use the absolute value 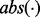 to capture positive and negative correlations indistinctively among genes, which improves the prediction performance in our test. Despite its simplicity, we consider this measure more suited than the traditional Pearson correlation coefficient (PCC) to measure co-expression in log-ratio expression data, in which each feature is a comparison in itself between two conditions. This can be more clearly seen by the following example: consider the log-ratio expression patterns of genes 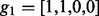 and 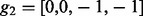. Analyzing these two patterns, we intuitively do not expect any relation between their corresponding genes because the expression of gene *g*_1_ is not affected at all when gene 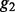 changes (i.e. 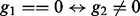) and vice versa. This is very well expressed by the cosine correlation, which returns a value 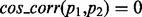. Contrarily, the PCC only considers the relative changes within the features of the patterns, which in this example are perfectly synchronized, thus returning a 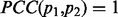, the opposite from what we expect.

In equation (1), 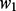 and 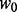 weight the influence of 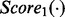 and 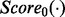, respectively; 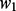 is defined by a function used to penalize expression signatures with a small number of features (details in Section 1 of Supplementary material), whereas 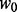 is a predefined parameter that allows us to adjust the level of discrimination of the expression signatures in order to avoid overfitting the training samples.

#### 2.3.1 Feature selection

The feature selection algorithm uses the *ESS* score in equation (1) to find a suitable expression signature for each positive gene 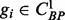. An exhaustive search, however, is not possible because it requires the evaluation of 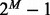 possible subsets of features for each positive gene. Consequently, we use an iterative and fast exploration scheme, referred as *signFS*, which uses suitable heuristics to efficiently search for discriminative expression signatures.

Given a gene expression pattern 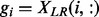, *signFS* starts by selecting an initial set 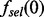 of features where gene *i* significantly changes its level of expression. We define as significant, a change with a FDR value <0.1 in 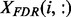. Afterwards, *signFS* performs an iterative process that, at each iteration *t*, adds and/or removes a suitable subset of features 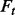, from 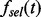. These changes must increase the expression pattern score 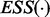. As a consequence, the new subset 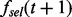 should provide better discriminative properties for gene function prediction. To favor the exploration of changes that increase discrimination, only a 20% of the total features, showing the lowest FDR and not in 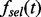, can be added at each iteration. This fosters the inclusion of features in 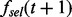 that show the most significant expression changes. Details about the scheme used to select subset 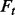 can be seen in Section 2 of Supplementary material. This iterative process continues until consecutive modifications of 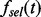 do not increase the respective score 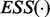.

### 2.4 Classification: using expression signatures to predict new gene associations

The aim of the classification scheme used by DLS is to predict new genes for a biological process of interest *BP*. Briefly, as expression signatures are discriminative, DLS considers that if a gene is highly co-expressed with the expression signature of a gene in 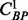, then it is likely to be involved in *BP*.

A relevant issue with respect to the previous classification scheme is that not all the expression signatures have the same potential to predict functional associations. In effect, this potential depends on several factors such as type of gene, type of biological process, level of noise in the data and biological complexity of interprocess co-regulations. DLS overcomes these issues by using a Bayesian inference approach that allows it to adaptively decide the minimum co-expression level needed by each signature to predict a gene with a given confidence. Consider a hypothesis, *h*, representing that an unknown gene 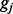 belongs to the positive class 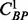. In addition, consider evidence, *e*, indicating that gene 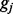 has a co-expression level, *L*, with respect to the expression signature of gene 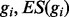. We can estimate the posterior probability 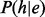 by using the Bayes rule: 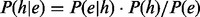.

Prior probability *P* (*h*) can be estimated directly from training data by calculating the proportion of positive versus negative genes in the training set. However, the estimation of the likelihood term 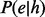 is not so straightforward as we need to estimate the probability density function of the co-expressions with respect to 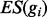. In this work, we estimate 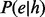 using a kernel-based density function estimation (Parzen, 1962). Given a data sample *x_i_* and a bandwidth σ, we use a Gaussian kernel function 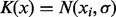, which measures the influence of sample *x_i_* in a location *x* of the input space. The bandwidth σ is a parameter that controls the smoothness of the density estimation and it is optimized using the cross-validation analysis described in Section 3.

Using the previous procedure, a gene 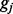 is predicted as positive by an expression signature *ES* (*g*_i_), if the co-expression *L* between them results in a confidence *P* (*h*|*e*) greater than a desired threshold. A graphical example of the above-mentioned procedure can be seen in Supplementary Figure S3.

### 2.5 Construction of a discriminative CN

One of the main features behind DLS is its ability to represent its predictions as a discriminative co-expression network (DCN), providing additional insights about the predictions and the biological process of interest. Formally, a DCN for a biological process *BP* is defined by a graph 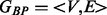, where vertices in set *V* represent genes, and edges in set *E* represent predictions from expression signatures to other genes. More precisely, there is an edge from gene 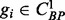 to gene 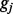, if there is an expression signature *ES* (*g_i_*) predicting that *g_i_* is related to *BP* with a confidence greater than a pre-defined threshold. In order to construct a DCN that involves all the genes related to *BP*, DLS applies the classification method to all the *N* genes in matrix *X*, including the ones in *C_BP_* used for training. This not only allows DLS to display a network description of the relations between training genes and predicted genes, but also to expose relevant relations among the positive genes, known to be involved in *BP*. A network description allows application of tools and concepts (Strogatz, 2001) developed in fields such as graph theory, physics and sociology that have dealt with network problems before (Alon, 2003). For example, a simple calculation of the node degree of the genes in the DCN can give relevant insights to discover central and highly coordinated genes in the biological process of interest.

### 2.6 Overcoming the FNs problem

One of the most relevant issues in using supervised learning methods to predict gene function is the FNs problem. In Section 2.2, we present a method to obtain an informative negative training set 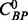. Unfortunately, due to the inherent complexity of gene behavior and the incompleteness of annotations, it is not possible to obtain a negative set without mislabeled genes, which may damage the prediction performance and evaluation.

To tackle the previous problem, we add to our training algorithm the option of a bootstrap step, which is able to automatically identify and temporarily discard from the set 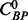, genes that are potential FNs. More specifically, this strategy is applied at the start of each iteration *t* of the feature selection algorithm *signFS*, performed in the training of each positive gene 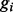. The strategy discards a negative gene 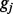 from iteration *t* if its co-expression with gene 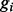, using the selected features in 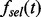, satisfies two conditions: (i) it has a value of at least 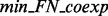 and (ii) it is among the top 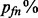 most highly co-expressed negative genes. Notice that these potential FN genes are not discarded permanently from the negative training set but they are only not considered in the evaluations of the patterns generated during step *t*. At the end of the training process, the method outputs the potential FNs detected by each expression signature.

The bootstrap option explained earlier in the text allows us to avoid overfitting problems due to the presence of FNs in the training set, however, it may affect the discriminative level of the signatures by ignoring some true negatives during the training process. Thus, we develop an iterative method, False-Negatives Discovery (FND), that takes advantage of this option in order to predict FN genes in a more precise and informative manner.

Initially, the list 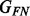 of potential FNs contains all negative genes in 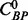 (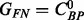). Then, each iteration of the method applies three consecutive steps, used to incrementally bound and refine the list 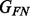. In the first step, a model is trained using the bootstrap option explained in the previous paragraph. 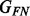 is then bounded to the potential FN genes detected by at least one trained signature. In the second step, the trained signatures are used to classify the genes in 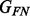, filtering out the ones not predicted as positive. Finally, in the third step, the training algorithm is used to search for a suitable expression signature for each gene in 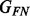. This algorithm is used without the bootstrap option. Then, a gene 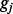 in 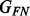 is predicted as a FN if the method is able to find an expression signature 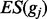 that satisfies two conditions: (i) 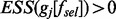 [equation (1)] and (ii) 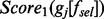 [equation (2)] is greater than the average 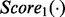 obtained among the valid expression signatures of the positive class 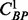. The first condition imposes to the predicted FNs to be discriminatively connected to other positive genes, whereas the second condition imposes them to be at least as connected as an average positive gene.

The three steps described earlier are executed iteratively by the FND method, automatically moving the predicted FNs to the positive set of the next iteration. The method stops if no new FNs are predicted or if a maximum number of iterations are reached. After performing the FND method, the training set can be refined, either by eliminating the predicted FNs from the negative set or by moving them to the positive set. This refined set is then used to train a DLS model and obtain the final predictions.

## 3 EXPERIMENTAL SETUP

A systematic evaluation was performed using an *A. thaliana* expression dataset and 101 GO biological processes. We compare the performance of DLS against two widely used state-of-the-art algorithms: SVMs (Brown *et al.*, 2000) and CNs (Vandepoele *et al.*, 2009).

In our tests, we used two expression datasets, pre-processed as described in Section 2.1, but using different procedures to define the control–test pairs of experimental conditions. In the first dataset, *M* = 643 pairs were manually defined by an expert, starting from a raw dataset containing 2017 *A. thaliana* ATH1 microarray slides (including replicates). We refer to this dataset as the ‘expert-dataset’. For the second dataset, a total of *M* = 3911 features were derived by the automatic procedure described in Section 2.1, starting from an updated raw dataset containing 3352 slides. We refer to this dataset as the ‘automated-dataset’. Most slides were obtained from the International Affymetrix Service of the Nottingham Arabidopsis Stock Centre (NASC, www.affymetrix.arabidopsis.info).

The evaluations consider the selection of 101 representative GO-terms from the 3500+ GO-terms available for *A. thaliana* in the biological process ontology. This selection was performed using the annotations available in GO on May 8, 2008. First, we filtered out all the annotations with IEA evidence code (Inferred from Electronic Annotation), as they are not reviewed by a curator. Then, we selected representative functional GO-terms using a depth-first strategy, searching for the first GO-term of each branch containing between 30 and 500 annotated genes. Thus, this selection is representative of the space of possible biological processes in the sense that all the branches of the GO DAG are represented by at least one GO-term in our selection. In other words, all the GO-terms that were filtered out are either subcategories (descendants) or broader categories (ancestors) of at least one of the selected GO-terms. The selected GO-terms are Levels 2–6 in the GO hierarchy and cover a wide range of biological processes, such as responses to different stimulus and various metabolic and developmental processes. The complete list of selected GO-terms is available in a Supplementary material spreadsheet file.

We derived a labeled training set for each selected GO-term, as described in Section 2.2. The number of positive genes in these training sets varies from 30 to 474, with an average of 162 genes. The number of negative genes varies from 1011 to 4112, with an average of 3105 genes. As can be seen, negative training sets are much bigger than positive ones, which is expected, because most genes are not involved in a particular biological process.

Cross-validation tests were performed using three 10-fold cross-validation tests over each GO-term. Each test was performed using a different 10-fold partition. As evaluation metrics we used:
(4)
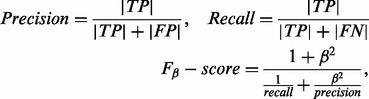

where 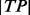, 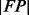 and 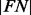 correspond to the number of true positives, false positives and false negatives, respectively. Precision measures the proportion of positive predictions that are correct. Recall measures the proportion of positive genes that are predicted as positive. Finally, the 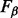 score provides a joint evaluation of both precision and recall, by calculating their harmonic mean. The 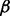 parameter controls the weight given to precision with respect to recall. In our tests, we used 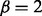 (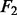 score), in order to favor accurate models over models with high recalls but large false positive rates.

In addition to the cross-validation analysis, an alternative, more realistic evaluation was performed, testing the enrichment of new annotations available on September 7, 2010, in the positive predictions of each method trained using the annotations of year 2008. This enrichment was tested using a hypergeometric distribution and a *P*-value threshold of 0.1 to consider enrichment.

**Fig. 1:**
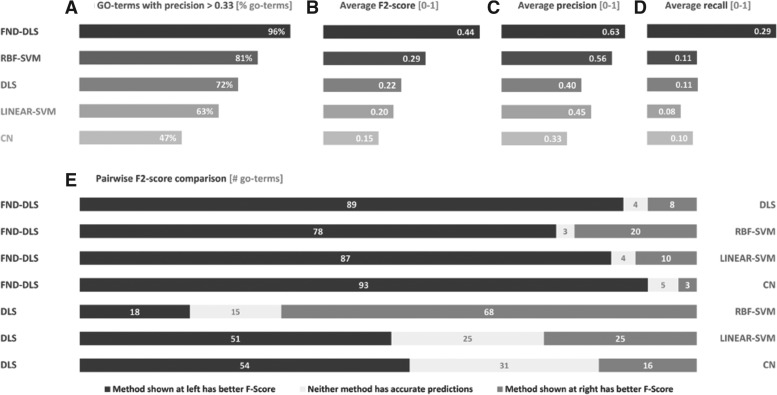
Results of the 10-fold cross-validation analyses performed over 101 representative GO biological processes. FND-DLS consistently shows the best overall performance, demonstrating the power of our method and the importance of handling false negatives (FNs) present in training sets. Despite FNs, all the tested supervised methods show superior prediction performances than the semi-supervised method CN. (E) shows the number of GO-terms in which one method attains better *F*_2_-scores than the other (details in Section 3).

**Fig. 2:**
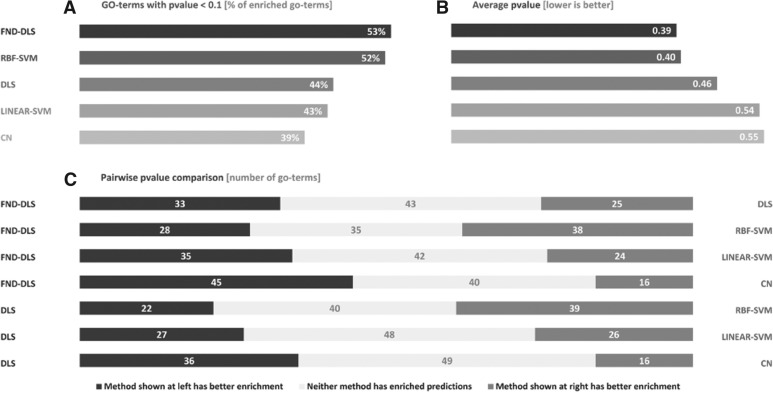
Summary of the results of an enrichment analysis performed over 101 representative GO biological processes. The analysis consists of testing the enrichment of new annotations from year 2010, in the predictions done by each method using annotation from year 2008. FND-DLS and RBF-SVM show the best overall performances, with a small advantage for FND-DLS. Figure (C) shows the number of GO-terms in which one method attains better enrichments (lower *P-*values) than the other (details in Section 3).

In order to facilitate the analysis of our results, we summarize them using three criteria. The first criterium consists of counting the number of GO-terms in which each method attains useful predictions. In the case of cross-validations, we consider as useful the GO-terms with precisions >0.33, meaning that at least one of three predictions are correct (Fig. 1A). In the case of enrichment analyses, we consider as ‘useful’ the GO-terms attaining enriched predictions (Fig. 2A). The second criterium consists of evaluating the average performances of the methods considering the 101 tested GO-terms. In cross-validations, we include precision, recall and 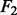-score averages (Figs 1B–D), whereas we include *P*-value averages for the enrichment analyses (Fig. 2). Finally, the third criterium consists of a pairwise comparison of the performances of the methods over each GO-term. Given two methods, A and B, we count the number of GO-terms in which A outperforms B and vice versa. Only GO-terms with useful predictions are counted. The 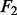-scores and *P*-values were considered as performance measures for cross-validations (Fig. 1E) and enrichment analyses (Fig. 2C), respectively.

The workflow used for the evaluations is as follows. We first perform cross-validation and enrichment analyses using the expert-dataset as described earlier in the text. The automated dataset is evidently more prone to both useless and redundant features, as some of them may be defined using biologically meaningless comparisons. Thus, the expert-dataset is used in order to ensure quality control–test condition pairs for the evaluations. In addition, we perform enrichment analyses using the automated-dataset with two specific aims: (i) test the potential of the automated-dataset for function prediction and (ii) test the performance of the methods in datasets with an increasing number of conditions (features). Thus, in addition to the complete dataset of *M* = 3911 features, we use two additional smaller datasets, defined by a random selection of *M* = {1000, 2000} features.

In terms of the evaluated methods, we use the configuration and parameters providing the highest average 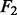-score in the cross-validation analysis. In the particular case of the proposed method, we report the performance of two alternative configurations, one using the False Negative Discovery method (FND-DLS), described in Section 2.6 and other without using it (DLS). In the case of FND-DLS, the final predicted FNs are added to the positive training set.

For the SVM method, we use the implementations available as part of the library for support vector machines (LIBSVM) (Chang and Lin, 2001). Similar to Brown *et al.* (2000), we tested four types of kernels: RBF, linear kernel and two polynomial kernels with degrees equal to two and three, respectively. As reported by Brown *et al.* (2000), RBF-SVM shows the best performance. However, as linear-SVM has the advantage of being more easily interpreted, it provides a good reference point to compare the performance of our method. Consequently, we report the results of both, RBF-SVM and linear-SVM. In terms of the selection of relevant parameters for the different SVM models, we tested different configurations following the default values suggested by the LIBSVM library and the optimization methods proposed by Brown *et al.* (2000). More details about the parameters selected for the SVMs can be found in Section 6 of Supplementary Material.

For the CN method, we construct the networks using the cosine correlation metric. Predictions are performed using a guilt-by-association criteria, using the hypergeometric distribution and Bonferroni correction for multiple tests. We tested five networks, applying correlation thresholds of 0.5, 0.6, 0.7, 0.8 and 0.9, respectively. In addition, we tested three *P*-value thresholds, 0.1, 0.05 and 0.01. We report the results of the CN model using a correlation and *P*-value value thresholds of 0.6 and 0.1, respectively, as this provides the highest average 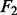-score.

The code and data to run these analyses over each method are available for MATLAB® programming software and can be downloaded from the link provided in the Availability Section.

## 4 RESULTS AND DISCUSSION

**Fig. 3:**
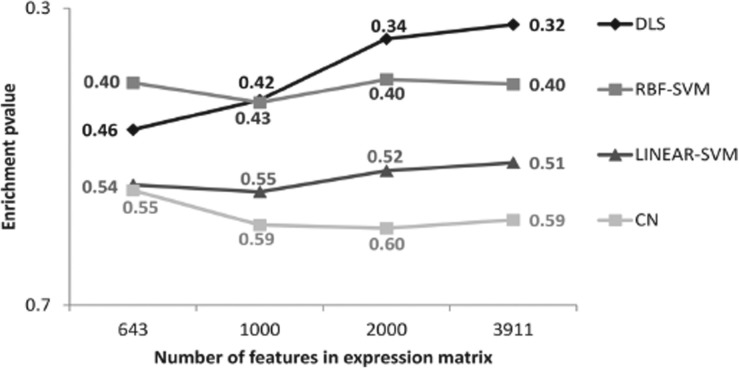
Results of enrichment analyses done over gene expression datasets with an increasing number of features (details in Section 3). In contrast to both SVM and CN, DLS shows a remarkable ability to systematically increase its performance when more features are added to the dataset. In fact, for the datasets with *M* ≥ 1000 DLS outperforms all other methods, showing the greatest potential to exploit the increasing amounts of gene expression data.

The results of cross-validation and enrichment analyses using the expert-matrix are summarized in Figures 1 and 2, respectively. The results of the enrichment analysis using the automated-matrix are summarized in Figure 3. The complete report of results can be found in Supplementary material spreadsheets available online. The rest of this Section presents and discusses these results.

### 4.1 FND-DLS shows the best overall prediction performance

Our results show that FND-DLS outperforms all competing methods, whereas RBF-SVM consistently attains the second best performance. In the case of the cross-validation analysis, FND-DLS attains useful predictions (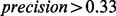) in 96% of the considered GO-terms, corresponding to 15%, 24%, 33% and 49% more GO-terms than RBF-SVM, DLS, linear-SVM and CN, respectively (Fig. 1A). In addition, it attains an average 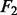-score of 0.44, whereas RBF-SVM, DLS, linear-SVM and CN attain averages equal to 0.29, 0.22, 0.20 and 0.15, respectively (Fig. 1B). Although FND-DLS attains better average precisions than the other methods (Fig. 1C), its supremacy in terms of the 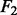-score is mostly explained by its higher recalls. FND-DLS attains an average recall of 0.29, whereas RBF-SVM, DLS, linear-SVM and CN attain average recalls equal to 0.11, 0.11, 0.08 and 0.10, respectively (Fig. 1D).

We see four main factor that may explain the overall small level of recalls obtained by the methods: (i) some genes may be regulated under experimental conditions not available in the expression dataset; (ii) some genes may not be regulated at a transcriptional level and thus, may not have (common) expression patterns; (iii) due to missing functional labels, some genes may only be regulated by (or regulate) genes not present in the positive training set and thus, it may be impossible for the methods to discriminate them and (iv) FNs genes may share and mask some discriminative patterns present among positive genes. The higher levels of recall achieved by FND-DLS over the other methods remark the importance of the last two factors described above.

The higher precisions obtained by FND-DLS supports the effectiveness of the FND process. The FND process iteratively moves the predicted FNs to the positive set. Thus, if FND-DLS wrongly predicted FNs, this genes would become false positive genes which, in turn, would decrease the precision of FND-DLS.

Cross-validation is useful to assess the relative performance of the methods; however, its results must be considered with caution (Varma and Simon, 2006). To tackle this, we use an enrichment analysis over a completely new set of labeled genes (i.e. new annotations from year 2010) to assess the performance of our method in an alternative and more realistic scenario (details in Section 3).

Interestingly, the results of the enrichment analysis confirm the supremacy of FND-DLS over RBF-SVM, although its overall advantage is smaller than in the cross-validation test (Fig. 2). FND-DLS attains enriched predictions in 53% of the GO-terms, whereas RBF-SVM, DLS, linear-SVM and CN attain enriched predictions in 52%, 44%, 43% and 39% of the GO-terms, respectively (Fig. 2A). Note that some GO-terms have few or no new genes annotated on year 2010 with respect to year 2008 and thus, it is very difficult or even impossible for the predictions to be enriched. In addition, the enrichment performance is affected by the same four factors exposed above for cross-validation. In terms of enrichment P-value (lower P-value represent higher enrichments), FND-DLS attains an average of 0.39, whereas RBF-SVM, DLS, linear-SVM and CN attain averages equal to 0.40, 0.46, 0.54 and 0.55, respectively (Fig. 2B).

### 4.2 Discriminative methods, DLS and SVM, provide more accurate gene function predictions than CNs

According to our experiments, both versions of DLS and SVM outperform CN. Although CN obtains similar average recall levels than SVMs and DLS (without FND), it fails in providing predictions as precisely as them (Fig. 1C and D). These results show the advantages of using discriminative training techniques in contrast to semi-supervised techniques in attaining accurate gene functional predictions. This assertion is further supported by the results of the enrichment analysis.

### 4.3 There is no method to rule them all

Although FND-DLS and RBF-SVM show the best overall performances, when comparing the performance at a term-by-term scale, we can only conclude that there is no method able to attain the best performance through all GO-terms (Figs. 1E and 2C). There are many factors that can bias the predictability of genes of a biological process toward one method or another. For example, in GO-terms related to responses, we see a bias in the predictability toward DLS in expense of CN, as the responses are usually expressed under specific environmental or physiological conditions, which DLS is able to detect due to its local search for discriminative features.

### 4.4 The discriminative and local expression patterns of DLS provide effective and meaningful predictions

According to the FDR matrix 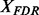, 96.2% of the expression changes in the log-ratio matrix 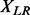 are not significant in the expert-dataset (considering an 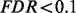 for significance), meaning that on average, genes show differential expression in only 24 (3.8%) of the 643 features. This sparseness emphasizes the importance of the selection of relevant features to achieve effective predictions, as the one performed by DLS. SVMs perform transformations to higher dimensions, which can also be interpreted as an implicit selection of relevant features. However, these transformations complicate the interpretation of the predictions and the extraction of further knowledge. Consequently, besides the prediction power of DLS, a key advantage over SVMs and other discriminative state of the art prediction methods is its ability to provide biologically meaningful and interpretable predictions while maintaining highly accurate predictions. Unlike SVMs, DLS is able to visually expose its predictions in the form of a network. This network delivers a much richer interpretability to the user than SVM, providing key information about the regulatory linkages that may exist between the genes of the functional class of interest. Finally, unlike both SVMs and CNs, DLS is able to explicitly reveal the experimental conditions and genes that are relevant for each prediction, by extracting the features and genes that define each expression signature.

### 4.5 DLS systematically improves its performance as more experimental conditions are added to the dataset

As stated above, the lack of informative features is one of the factors that may affect the prediction potential of the methods. In this sense, the increasing amount and variety of gene expression experiments represent both an opportunity and a challenge. If the number of available experiments increases, chances to find informative features among them also increase. However, the amount of uninformative and redundant features should also increase, adding extra noise that must be correctly handled by the prediction methods.

The results of the enrichment analyses performed using the automated-dataset support our previous hypothesis and one of the most remarkable features of our method (Fig. 3). When using the expert-dataset, containing 643 features, DLS achieves an overall P-value of 0.46. Interestingly, when using the automated-datasets, containing 1000, 2000 and 3911 features, its average P-value improves to 0.42, 0.34 and 0.32, respectively. In contrast, RBF-SVM is not able to improve its performance, linear-SVM shows little improvement, and the performance of CN even gets deteriorated. Notice that when using the automated-dataset, DLS achieves the highest overall performance in terms of enrichment, even without using the FND procedure.

These results show that DLS is able to overcome the underlying noise added by the automated-dataset by effectively extracting relevant and informative features. In addition, they support the usefulness of our automatic procedure to generate log-ratio expression datasets from poorly annotated experiments. But, most remarkably, they suggest that DLS should be the most benefited method as, in the future, more microarray experimental data becomes available.

## 5 CONCLUSION

In this work, we described DLS, a novel method that combines supervised machine learning and co-expression approaches to effectively predict new genes involved in a biological process of interest. We introduced four key concepts that allow DLS to effectively predict gene function: the derivation of informative training sets of genes by discovering FN training genes, the supervised search of discriminative expression patterns in subsets of experimental conditions (expression signatures), a Bayesian probabilistic approach to derive the confidence for each prediction and the construction of discriminative CNs to represent predictions.

By using an *A. thaliana* expression dataset and 101 GO biological processes, our experiments showed that DLS is able to provide effective gene functional predictions, with accuracies comparable to the highly discriminative SVMs, while maintaining the expressiveness of CNs. Interestingly, they also show that, unlike SVMs and CNs, DLS systematically improves its prediction performance as more experimental conditions are added to the dataset. Thus, we believe that the supervised use of co-expression proposed in this work opens new opportunities to extract meaningful biological hypothesis from the increasing amounts of expression data, and therefore, to cope with the need to understand gene functions and biological processes.

*Funding*: This research was funded by International Early Career Scientist program from Howard Hughes Medical Institute, Fondo de Desarrollo de Areas Prioritarias (FONDAP) Center for Genome Regulation (15090007), Millennium Nucleus Center for Plant Functional Genomics (P10-062-F), Fondo Nacional de Desarrollo Científico y Tecnológico (1100698), Comisión Nacional de Investigación Científica y Tecnológica-ANR program (ANR-007) and Corporación de Fomento de la Producción Genome Program (CORFO07Genoma01).

*Conflict of Interest*: none declared.
